# Autologous hematopoietic bone marrow and concentrated growth factor transplantation combined with core decompression in patients with avascular necrosis of the femoral head

**DOI:** 10.25122/jml-2022-0342

**Published:** 2023-01

**Authors:** Nasser Ghaly Yousif, Adnan Essa Kadhum Al Kilabi, Karrar Kareem Hatem, Hamdullah Hadi Al-Albaseesee, Wejdi Abbass Yassin Al-Fatlawy, Majid Alhamadani, Ulrich Aran Nöth, Ahmed Altmimi

**Affiliations:** 1 Department of Medicine, Medical College, Al Muthanna University, Samawah, Iraq; 2 Department of Surgery, Medical College, Kufa University, Kufa, Iraq; 3 Department of Surgery, Medical College, Jabir Ibn Hayyan Medical University, Najaf, Iraq; 4 Department of Surgery, Ministry of Health, Al Muthanna, Iraq; 5 Department of Regenerative Research, College of Medicine, Colorado University, Boulder, Colorado; 6 Department of Biology, Ministry of Health, Al Muthanna, Iraq

**Keywords:** avascular necrosis of the femoral head, core decompression, autologous hematopoietic bone marrow transplantation, concentrated growth factor, ANFH – avascular necrosis of the femoral head, CGF – concentrated growth factor, CI – confidence interval, CT – computed tomography, HIV – human immunodeficiency virus, VAS – visual analogue scale, WOMAC – Western Ontario and McMaster Universities Osteoarthritis Index

## Abstract

The study aimed to assess the effectiveness of autologous hematopoietic bone marrow and concentrated growth factor (CGF) transplantation and core decompression in patients with avascular necrosis of the femoral head (ANFH). We performed a single-center prospective study on 31 patients with non-traumatic early-stage (stage I to III) ANFH based on the 1994 classification of the Association Research Circulation Osseous (ARCO). The patients were subjected to bone marrow aspiration from the posterior iliac crest, separation, and concentration of growth factors from the bone marrow aspirate, core decompression of the femoral head, and injection of hematopoietic bone marrow and CGFs into the necrotic lesion. Patients were evaluated using the visual analogue scale, the WOMAC questionnaire, and X-ray and MRI examinations of the hip joints before, at 2, 4, and 6 months after the intervention. Patients had a mean age of 33 years (range 20–44 years), 19 (61%) of them being male and 12 (39%) females. The presentation of the disease was bilateral in 21 patients and unilateral in 10 patients. The main cause of ANFH was steroid treatment. The mean VAS and WOMAC scores were 48.37 (SD: 14.67) out of 100, and the mean VAS pain score was 50.83 out of 100 (SD: 20.46), respectively, before transplant. This value significantly improved to 22.31 (SD 12.12) of 100, and the mean VAS pain score was 21.31 of 100 (SD: 20.46) (P=0.04). MRI showed a significant improvement (P=0.012). Our results suggest that autologous hematopoietic bone marrow and CGFs transplantation with core decompression have a beneficial effect in early-stage ANFH.

## INTRODUCTION

Avascular necrosis of the femoral head (ANFH) is a debilitating condition characterized by progressive necrosis of bone tissue caused by an increase in intraosseous pressure and a reduction in the blood supply of the femoral head, with unknown pathogenesis [1]. ANFH, also called osteonecrosis, ischemic necrosis, or aseptic necrosis, has an incidence of 20,000–30,000 cases per year, with approximately 300,000–600,000 people being affected in the United States [2]. The most common non-traumatic cause is glucocorticoid treatment, with ANFH occurring in 5% to 40% of patients receiving long-term glucocorticoids [3]. Smokers are at an increased risk of developing ANFH. A study found that smokers had a 2.5 times higher risk of developing AFNH than those who did not smoke. Furthermore, current smokers had a higher risk of developing ANFH (OR 2.53; 95% CI:1.68–3.79) [4]. Long-term alcohol intake also increases the risk of ANFH. The average duration of alcohol abuse until the diagnosis of ANFH is 9.5 years, and the majority of people who drink excessively and develop ANFH are over 50 years old and consume more than 400 mL of alcohol a week [5].

Early treatment, prior to the subchondral collapse of the bone in the late stages of ANFH, is essential to conserve the structure and function of the joint and prevent the need for total hip replacement, a common treatment option for terminal ANFH [6]. According to recent findings, ANFH is linked to a lower level of osteoprogenitor cells in the bone marrow of the femoral head [7, 8]. Studies have also revealed a potential benefit after the implantation of bone marrow containing osteogenic precursors into the necrotic lesions [9]. Bone marrow-derived mesenchymal stem cells (BMSCs) differentiate into various cell lineages of mesodermal origins, such as bone, cartilage, muscle, and adipocytes [10–13]. Red bone marrow includes not just hematopoietic elements but also stem cells containing osteogenic bone cell precursors. In 1869, Goujon discovered that bone marrow has the ability to stimulate bone formation in rabbits [14]. Therefore, bone marrow is frequently used in the treatment of fracture non-union, as well as to improve the osteogenic effects of bone grafts and their alternatives [15]. It is widely assumed that osteogenic cells are derived from stem cells found in the stroma of bone marrow, and bone marrow transplants are a valuable source of osteogenic precursor cells [16]. BMSCs are simple to isolate and culture and are also found in various tissues, including the synovial tissue, adipose tissue, and umbilical cord blood. According to several recent studies, the number of BMSCs and their osteogenic differentiation abilities are impaired in individuals with corticosteroid- and alcohol-induced femoral head osteonecrosis [17–23].

## MATERIAL AND METHODS

We conducted a single-center prospective analytical study in a private hospital in Iraq on 31 patients with early-stage (stages I to III) non-traumatic ANFH. Baseline demographic data included age, gender, etiological factors, and ANFH stage according to the Ficat and Arlet classification based on plain radiograph, MRI, and the location of the necrotic lesion in each hip. A prospective, randomized, open-label was conducted involving patients who were developing ANFH at Iraqi private hospital, Iraq, from January 31, 2021, to November 2022. The intervention group received decompression and autologous bone marrow concentration (n=31).

The stage of ANFH was determined based on the Association Research Circulation Osseous (ARCO) classification 1994 for early-stage osteonecrosis, as follows:

- **Stage 0:** Normal plain radiograph, normal computed tomography (CT), normal magnetic resonance imaging (MRI) or scintigraphy;
- **Stage I:** Normal plain radiograph with abnormal CT or MRI;
- **Stage II:** Plain radiograph: trabecular bone changes without variations in subchondral bone; MRI: unusual and diagnostic appearance;
- **Stage III:** Plain radiograph: a highlight on the signs of a subchondral fracture displaying the "crescent sign";
- **Stage IV:** Radiographic manifestation of femoral head flattening;
- **Stage V:** Plain radiograph reveals a sign of femoral head flattening in addition to osteoarthritic variations, including decreased joint space and acetabula alteration;
- **Stage VI:** It appears that the entire joint is destroyed.

### Study procedures

All study participants were subjected to the study procedures consisting of the four steps below:

1. Blood samples were taken to perform a complete blood count and assess the presence of virus markers for HIV, hepatitis B, and hepatitis C.
2. Isolation of growth factor concentrated (GFC). The entire procedure was completed in a centralized clean room that met the Good Manufacturing Practices (GMP) requirements. Forty milliliters of whole blood were drawn from each patient in a vacutainer containing acid citrate dextrose (ACD) to prevent clotting. Also, 5 ml of blood was drawn in an EDTA vacutainer for plasma collection. ACD vacutainer containing whole blood was centrifuged at 400 gravitational forces (g) for 15 minutes. The upper layer of plasma rich in platelets (PRP) was removed in another sterile centrifuge tube, leaving behind a buffy coat in the middle and packed red blood cells at the bottom. PRP was centrifuged again at 3000 g for 10 minutes.
  The supernatant containing platelet-poor plasma (PPP) was then removed and collected in other sterile centrifuge tubes for subsequent use. The platelet-rich pellet at the bottom was then suspended in 5 ml of plasma to dilute platelet-rich suspension using PPP. The final product was sterile-filtered through a 0.22 µm filter [24].
3. Hematopoietic bone marrow and stem cell concentrate were harvested through bone marrow aspiration from the posterior iliac crest of each patient. A small incision was made in the region of the posterior iliac crest, and a bone marrow aspiration needle was inserted between the posterior crest cortical walls. A total of 150–200 mL of bone marrow aspirate was obtained, which was transferred to a sterile pouch containing anticoagulants such as acid-citrate-dextrose and sodium citrate. The aspirated bone marrow specimen was washed under sterile conditions through class 2 laminar flow and filtered to eliminate lipids and clot particles. Then, 6 mL of concentrated red bone marrow was produced from 60 mL of the specimen using a Biomet Biologics system. Polynucleotide cells were isolated at 100 mL/min flow for about 50 seconds. Following the separation of mononuclear cells containing the stem cells, a cell count was performed. If the mean cell count of concentrated mononuclear cells was at least 2.5 million per mL, the cells were returned to the operating room in a sterilized plastic bag containing anticoagulants for injection. The entire procedure lasted less than an hour.
4. Core decompression: the patients were placed in a supine position on the operating table and anesthetized with either epidural or general anesthesia, depending on the anesthetist's decision and the patient's preference. A 1.5–2 cm incision was made at the level of the greater trochanter. Under C-arm X-ray guidance, a cannulated drill was used to insert a Kirschner wire (K-wire) into the center line of the trochanter towards the necrotic area. The end of the K-wire was implanted into the subchondral bone, 2–3 mm from the articular cartilage. Then, the K-wire was removed, and a bone marrow aspiration needle was inserted through the K-wire groove. Concentrated autologous hematopoietic bone marrow and CGFs were injected through the needle with light compression at the incision site and closed afterward with a silk suture.

### Outcome measurement and follow-up

The primary outcomes were pain on movement, assessed using the visual analogue scale (VAS), and hip function, assessed using the Lequesne index for knee osteoarthritis and the WOMAC Osteoarthritis Index. MRI scans were performed on 1.5 Tesla MRI scanners (Optima and Signa, General Electric Medical Systems, Milwaukee, Wisconsin, USA) with dedicated coils and routine avascular necrosis protocol, including coronal T1- and STIR or T2-weighted fat-saturated, axial T2-weighted fat-saturated and sagittal T1-weighted sequences. Approximately 60% of the MRI scans included an i.v. contrast coronal T1-weighted fat-saturated sequence. The size of the AVNFH lesion was evaluated in the T1-weighted coronal plane (greatest length and depth of the lesion and the freehand drawn area) and the T1-weighted sagittal plane. The areas of the necrotic lesion and the femoral head were freehand drawn from each image in the coronal plane, and then the sum of the area of the necrosis was divided by the sum of the area of the femoral head to determine the percentage of the volume of the AVNFH. Subsequently, lesions were graded as small (<15%), moderate (15–30%), and large (>30%), and the location of the necrotic lesion in the femoral head was estimated. The extent of the necrotic lesion to the weight-bearing area of the femoral head was estimated according to Japanese Investigation Committee (JIC) guidelines [24].

The patients remained in the hospital overnight after the intervention and were discharged with movement restriction for seven days, then free movement with restriction from climbing stairs, restricting climbing stairs for 3 months. All patients were evaluated at 2, 4, and 6 months after the intervention, using the same assessment as before the intervention.

### Statistical analysis

Continuous variables were compared with the paired t-test and non-parametric data with the Wilcoxon rank test. We used ANOVA for continuous data and Friedman's test for non-parametric data to assess the effect of the intervention on outcomes during follow-up. The significance threshold was set at p<0.05. All analyses were done with SPSS version 16 (SPSS for Windows, Version 16.0. Chicago, SPSS Inc.).

## RESULTS

The mean age of the patients was 33 years (range 20–44 years), 19 (61%) of them being male and 12 (39%) females. The ANFH stage classification is presented in Table 1. The presentation of the disease was bilateral in 21 patients and unilateral in 10 patients (Table 2). The main cause of ANFH was steroid treatment (Table 3).

**Table 1:** ANFH stage classification according to Steinberg.

**0**	Pain: no symptoms; MRI: non-diagnostic; X-ray: normal.
**1**	Pain: mild pain occurs with internal rotation in the affected hip; MRI: diagnostic; X-ray: normal.
**3**	Pain: worse pain or persistent duration increasing sclerosis or cysts within the femoral head.
**4**	Collapse of subchondral region results in a "crescent sign".

**Table 2:** Demographic characteristics of the study population.

Age groups	Patients		Unilateral ANFH		Bilateral ANFH	
	n	%	n	%	n	%
**20–25 years**	10	32.25	3	9.6	7	22.5
**26–31 years**	12	38.7	5	16.12	7	22.5
**32–37 years**	6	19.35	1	3.2	5	16.12
**38–44 years**	3	9.6	1	3.2	2	6.4

**Table 3:** Distribution of etiologic factors of AFNH in the study population.

Etiologic factors	n	%	P-value<0.05
**Steroids**	22	72%	0.02
**Idiopathic**	7	23%	0.04
**Smoking**	2	5%	0.1
**Alcohol**	0	0	-
**Sickle cell disease**	0	0	-

**Figure 1:**
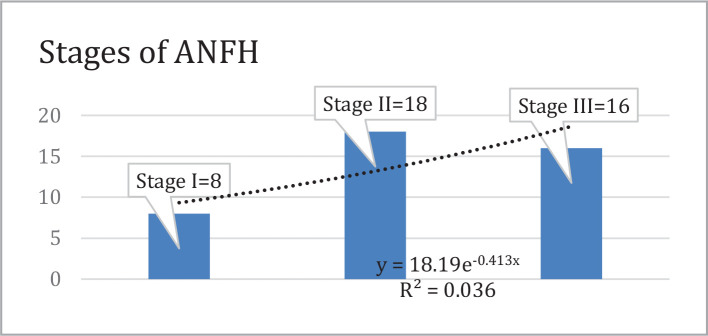
The stages of ANFH, according to Steinberg's classification.

**Figure 2:**
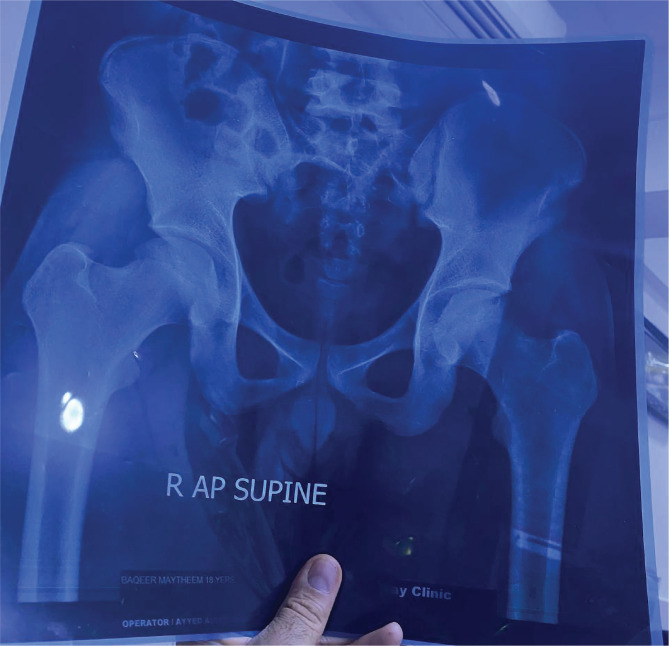

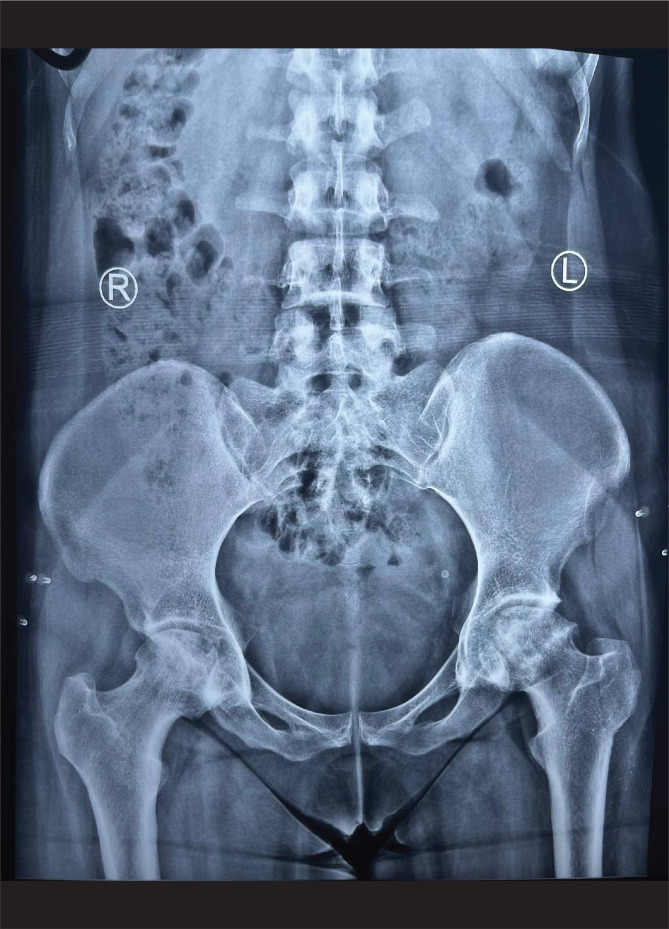

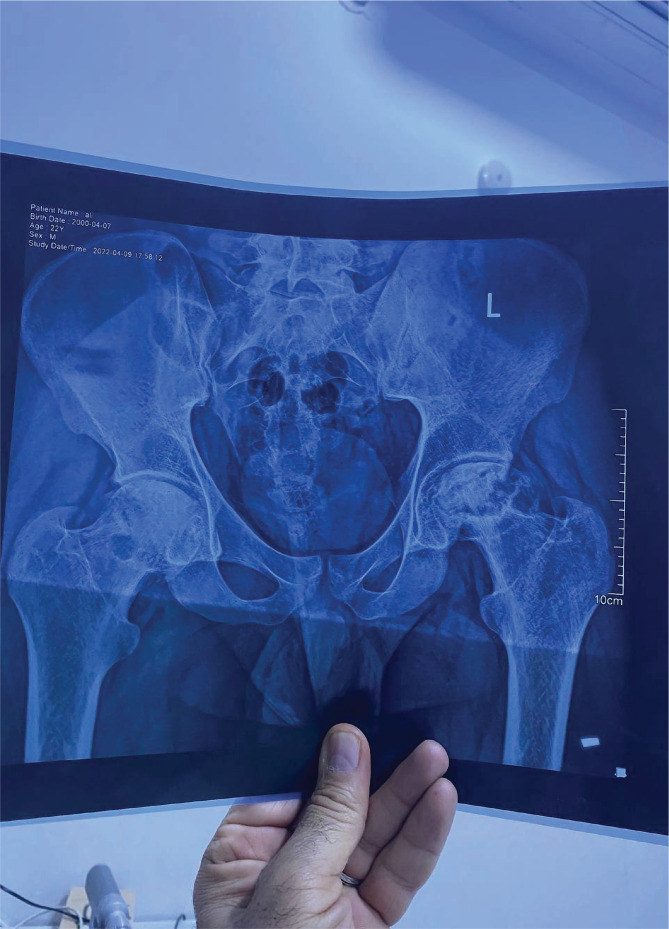
Preoperative hip joint X-ray showing ANFH of different stages.

Based on the ARCO classification 1994, at baseline, 8 (26%) patients had ANFH stage I, 18 (58%) patients had ANFH stage II, and 5 (16%) patients had ANFH stage III (Figures 1 and 2).

**Figure 3:**
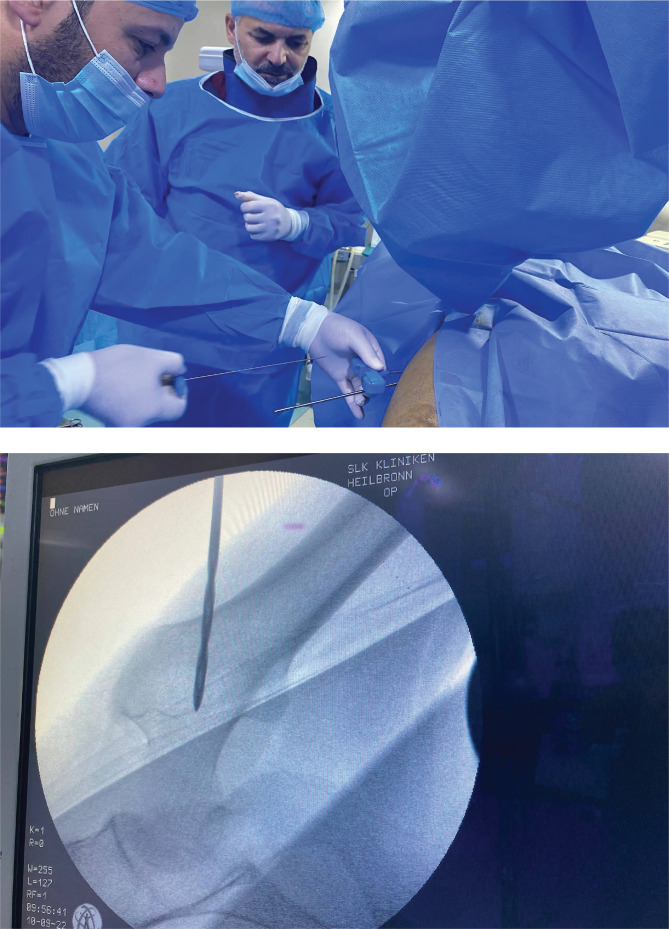

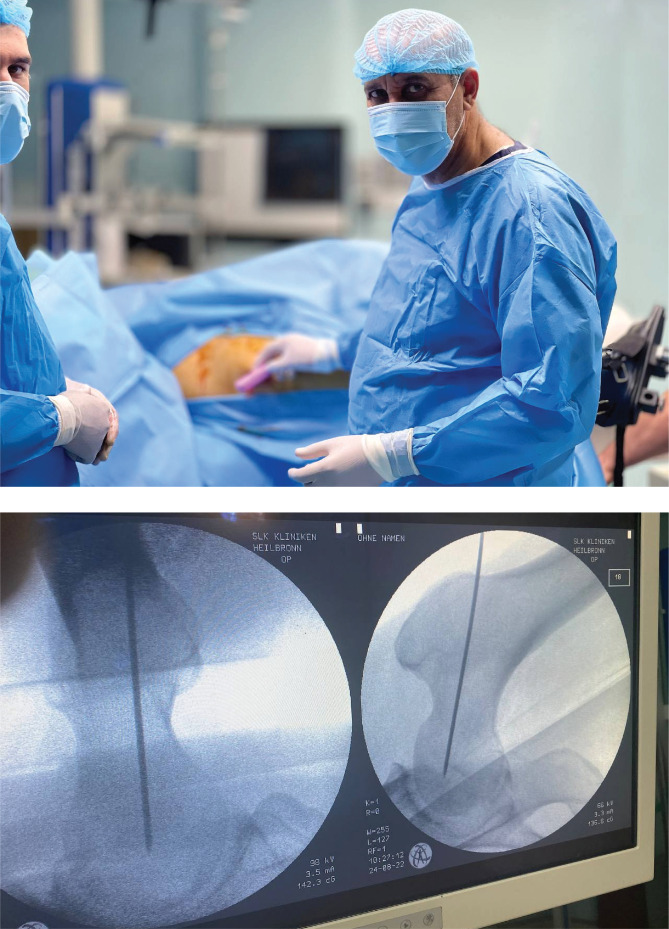
Bone marrow harvesting, stem cell processing, and reinjection into the femur head through drilling.

The surgical intervention, performed under general or spinal anesthesia, included the collection of bone marrow, processing of stem cells, and re-injection of the stem cells into the hip bones (Figure 3).

During the intervention, oxygen saturation, heart rate, and blood pressure were stable in all patients, and there were no complications such as pulmonary embolism, thrombophlebitis or intertrochanteric fracture. Postoperative complications occurring in the follow-up period are presented in Table 4.

**Table 4:** Complications observed during follow-up.

Complication	n			%	P-value<0.05
	2 months	4 months	6 months		
**Pain during movement**	11	3	0	45	0.04
**Limping**	7	2	1	32	0.93
**Fever**	0	0	0	0	-
**Infection**	0	0	0	0	-

**Figure 4:**
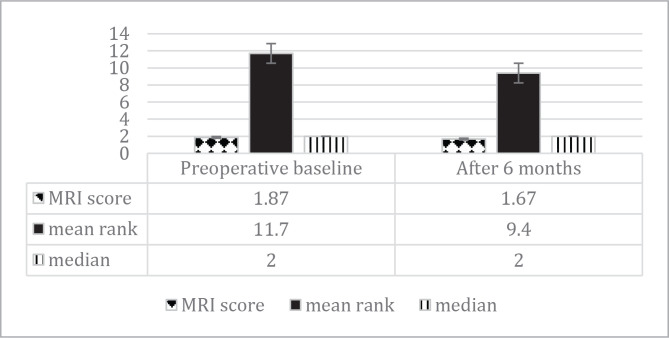
Preoperative and postoperative MRI scores.

**Figure 5:**
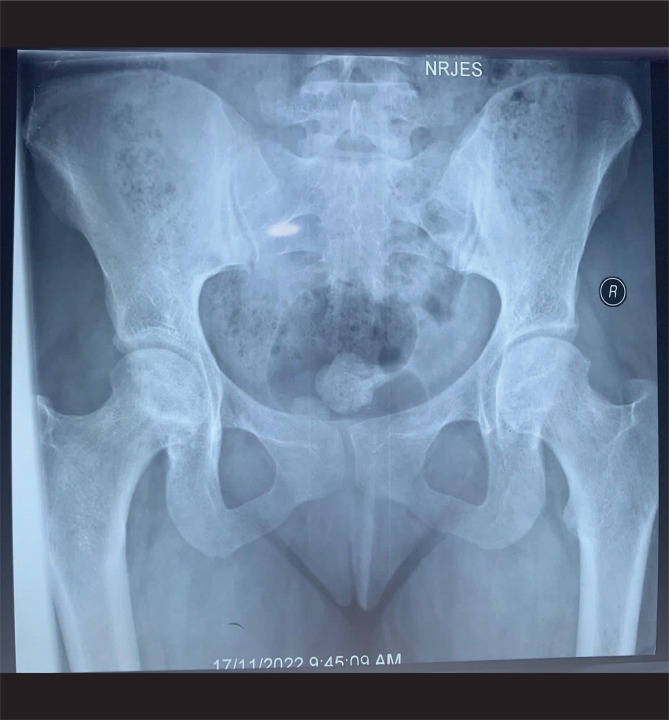

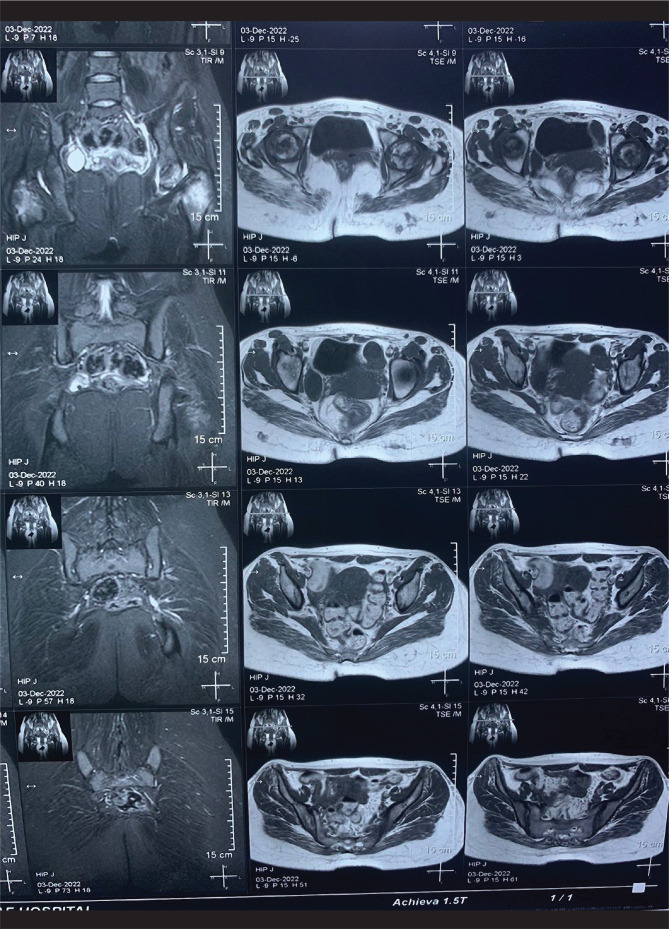

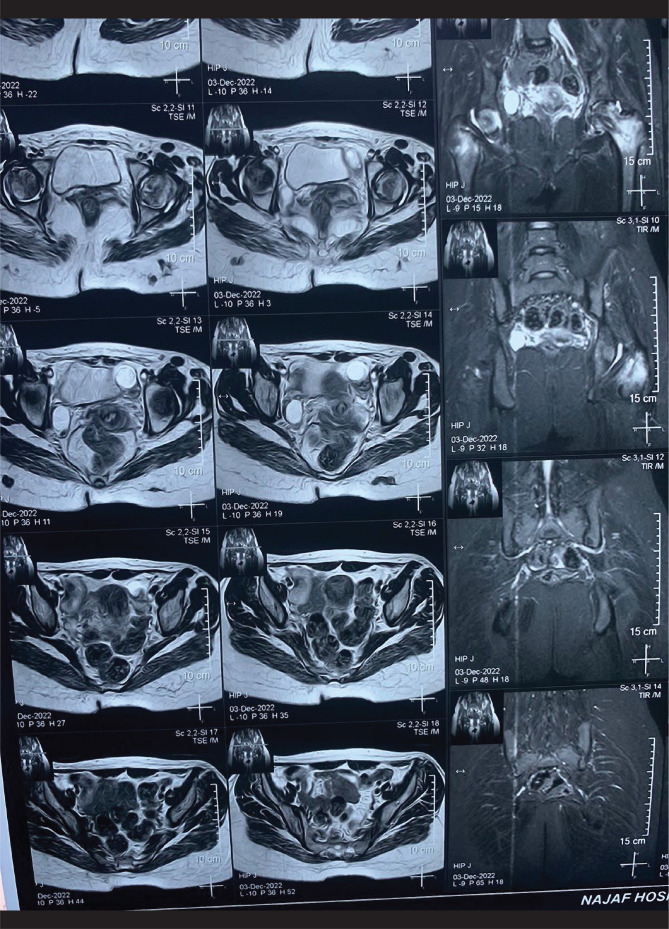

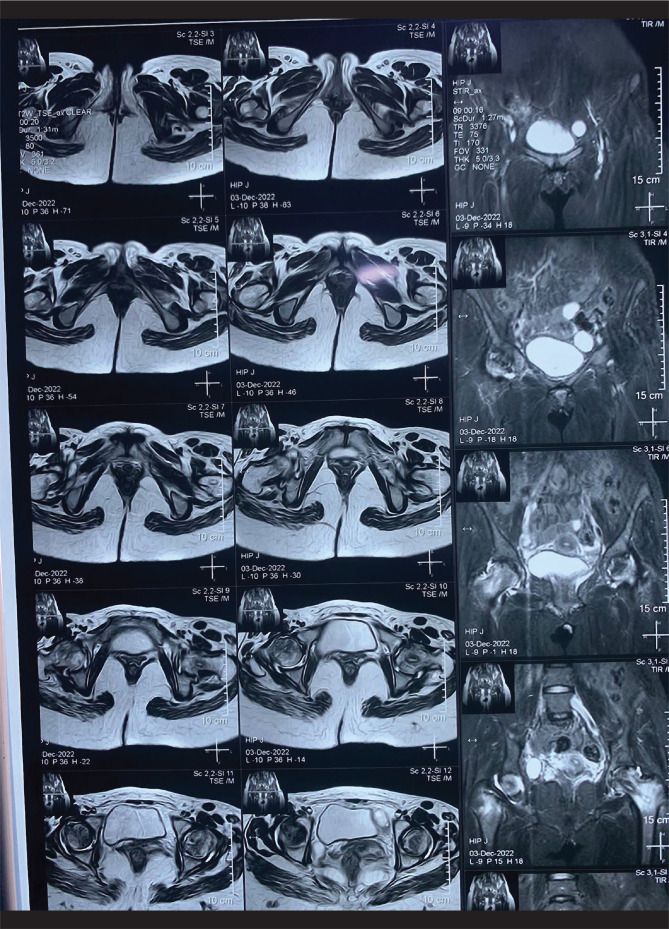
MRI changes in the follow-up period.

WOMAC Osteoarthritis Index scores improved from 33.4±2.5 at baseline to 10±1.2 at 6 months (p=0.012). Changes in WOMAC scores during the follow-up period are presented in Table 5, while MRI changes are presented in Figures 4 and 5.

**Table 5:** WOMAC index at baseline and during follow-up.

Time	Score (mean±SD)	P-value<0.05
**Baseline**	33.4±2.5	0.012
**2 months**	22.6±2.9	
**4 months**	17.4±2.3	
**6 months**	10±1.2	

## DISCUSSION

Several theories about the pathogenesis and etiology of ANFH have led to the development of different therapeutic methods with varying efficacy [25–27]. Some of these theories include the lipid metabolism theory, the MSC differentiation function theory, the intravascular coagulation theory, the cell apoptosis theory etc [28]. The purpose of the present study was to evaluate the clinical outcomes of autologous hematopoietic bone marrow and CGF transplantation combined with core decompression in patients with ANFH.

One of the most widely utilized therapies for ANFH is core decompression, which requires drilling into the necrotic areas to increase blood supply and decrease edema. Core decompression can also decrease pain and promote capillary regeneration, but it does not entirely solve the issue of femur repair [29]. In 2002, Hernigou and Beaujean described a method for injecting MSCs into the necrotic region in conjunction with core decompression [30].

More than one-third (38.7%) of the patients in our study were in the 26–31 age group, while patients aged 36–44 years accounted for only 9.6% of the study population. These findings are similar to those in other studies [31–33], suggesting that ANFH affects mostly younger patients. The main causes of ANFH in our study were steroid treatment (72%), idiopathic (23%), and smoking (5%), consistent with other studies that have shown that steroids and smoking are the main etiological factors in the development of AFNH [33].

In the study of Yan Z *et al*. [34], the combination of autologous stem cell transplantation and core decompression resulted in progressive pain relief and improved hip joint function at the 2-, 4-, and 6-month follow-up. In another study, this combination was also able to slow the progression of ANFH, particularly when treatment was started as early as possible, in stages I and II [35, 36]. Another study found that the transplantation of autologous BMSCs significantly reduced pain and disease progression in patients with ANFH over a follow-up period of 5 years [37]. Similar results were obtained in another study, which showed that none of the lesions progressed in size or to a more advanced stage [38].

Our results showed that WOMAC scores improved significantly from 33.4±2.5 at baseline to 10±1.2 at 6 months (p=0.012). In a retrospective study on the effectiveness of concentrated BMSCs in ANFH, 94 of 534 (17.6%) cases have progressed to total hip arthroplasty after an average follow-up of 12 years [38]. On MRI, these patients showed significantly higher Harris Hip Score values and a smaller necrotic lesion. Also, 69 (18%) patients had total resolution of their necrotic lesion [15]. The study also observed that the number of injected stem cells had a direct effect on the healing of necrotic lesions in the femoral head [38]. Thus, in order to increase the number of injected stem cells, some studies have attempted the ex *vivo* amplification of iliac crest stem cells [39–41].

The main limitations of our study are the small sample of 31 patients and the short follow-up period of 6 months. A follow-up of at least 5 years would be needed to assess the effectiveness and long-term benefits of this treatment in ANFH.

## CONCLUSION

This study found that autologous hematopoietic bone marrow and CGF transplantation in combination with core decompression is effective for the treatment of early-stage non-traumatic ANFH. Our findings demonstrate the benefits of concentrated bone marrow stem cells in the regeneration of necrotic areas in the focal bone. Larger studies are needed to evaluate the effectiveness of current therapeutic methods in ANFH.
